# Antibiotic use appropriateness and its determinant factors among pediatric pneumonia patients in selected comprehensive specialized hospitals in Northwest Amhara, 2024: a prospective follow-up study

**DOI:** 10.1186/s12879-026-13447-8

**Published:** 2026-04-28

**Authors:** Edmealem Minlarg, Bekalu Kebede, Belayneh Yitayew, Tigist Tesfaye Tilahun, Tirsit Ketsela Zeleke, Teferi Bihonegn, Habtamu Molla Gietie, Fasil Tadesse Ferede, Sumeya Tadesse

**Affiliations:** 1 Department of Pharmacy, College of Health Sciences, Debre Markos University, Debre Markos, Ethiopia; 2 Department of Pharmacy, College of Health Sciences, Wachamo University, Hosahna, Ethiopia; 3 Department of Pharmacy, College of Health Sciences, Debre Tabor University, Debre Tabor, Ethiopia; 4 Department of Medical Laboratory Science, College of Medicine and Health Science, Debre Markos University, Debre Markos, Ethiopia; 5 Department of Pharmacy, School of Pharmacy, College of Medicine and Health Science, University of Gondar, Gondar, Ethiopia; 6 Department of Pediatrics, School of Medicine, Debre Markos University, Debre Markos, Ethiopia

**Keywords:** Appropriateness, North Amhara, Pneumonia, Antimicrobial stewardship

## Abstract

**Introduction:**

Proper use of antibiotics is the gold standard for treating bacterial pneumonia. Inappropriate prescribing leads to poor clinical outcomes and accelerates antibiotic resistance.

**Objectives:**

This study aimed to assess the appropriateness of antibiotic use and identify its determinants in pediatric pneumonia patients in selected hospitals in Northwest Amhara in 2024.

**Methods:**

This prospective multicenter study was conducted from June to September 2024 at three specialized hospitals in Northwest Ethiopia (Gondar, Debre Markos, and Bahir Dar). Data regarding diagnostic methods, pneumonia classification, and antibiotic regimens were extracted from the medical records of pediatric patients using a validated, structured checklist. The appropriateness of antibiotic use was evaluated against the 2021 Ethiopian National Standard Treatment Guideline and Infectious Diseases Society of America (IDSA) guidelines based on drug selection, dosage, frequency, and duration. Statistical analysis was performed using SPSS version 26. Multivariable logistic regression was employed to identify independent determinants of inappropriate antibiotic use, with statistical significance defined as *P* < 0.05.

**Result:**

Among the 360 included patients (median age 1.5 years; 58.9% male**)**, the majority (74.7%) were aged under five years, and 88.9% were diagnosed with Community-Acquired Pneumonia (CAP). Ceftriaxone was the most commonly prescribed antibiotic overall (38.1%). The prevalence of inappropriate antibiotic use was 38.3% (95% CI: 33.3–43.6%), with incorrect drug selection (42.9%) being the primary reason for inappropriateness. Independent predictors of inappropriate antibiotic use were prescriptions by pediatricians (AOR = 5.008, 95% CI: 1.228–20.425; *p* = 0.025), pediatric residents (AOR = 4.076, 95% CI: 2.492–6.667; *p* < 0.001), and the presence of comorbidity (AOR = 1.717, 95% CI: 1.062–2.777; *p* = 0.027).

**Conclusion:**

Inappropriate antibiotic use remains a major concern in managing pediatric pneumonia, contributing to poor clinical outcomes. Prescriptions by pediatricians and pediatric residents, and comorbidity were associated with inappropriate antibiotic use, and hence practitioners should never disregard these factors to prioritize those cases coming with them.

**Supplementary Information:**

The online version contains supplementary material available at 10.1186/s12879-026-13447-8.

## Background

Pediatric pneumonia remains a leading cause of morbidity and mortality worldwide [1]. Antibiotics serve as the primary treatment for bacterial pneumonia, with empiric therapy commonly initiated based on the likely pathogens, clinical setting, risk of multidrug-resistant (MDR) organisms, and other patient-specific risk factors [2, 3]. In pediatric pneumonia patients, guideline recommendations support the use of third-generation parenteral cephalosporin for unimmunized, or those in areas with penicillin resistance, or in severe cases, while combination therapy with macrolides, along with a β-lactam antibiotic, is recommended when Mycoplasma pneumonia and Chlamydophila are suspected [4, 5]. If clinical, laboratory, or imaging findings suggest a possible Staphylococcus aureus infection, vancomycin or clindamycin (selected based on local susceptibility data) should be added to the β-lactam therapy [3].

Appropriate antibiotic use involves selecting the correct drug, dose, frequency, and duration to meet patients’ unique needs [6]. However, failure to meet any of these elements leads to inappropriate use, which is a major driver of the emergence of antibiotic resistance [7, 8]. The improper utilization of medications presents a significant worldwide challenge [9]. Globally, more than half of the medications are used inappropriately, with studies showing that inappropriate antibiotic use for pneumonia has been estimated to be between 14.1% and 90.2% [6, 10–13].

The drivers of inappropriate antibiotic use are multifactorial. Key determinants include inadequate provider training, diagnostic uncertainty, poor adherence to clinical guidelines, and the over-prescribing of broad-spectrum antibiotics [14–17]. These issues are often exacerbated by systemic challenges, such as a lack of evidence-based policymaking and external pressures on prescribers [18]. Furthermore, practices such as prescribing multiple or unnecessary antibiotics, exceeding recommended dosages, and seeking prescriptions from multiple doctors worsen the issue [19, 20].

The impact of inappropriate antibiotic use is profound. It is the primary driver of the global antimicrobial resistance (AMR) crisis, increased healthcare costs, prolonged hospital stays, treatment failure, and mortality [21–23]. While antimicrobial stewardship programs have proven effective in improving adherence to guidelines, their implementation remains inconsistent [21, 24–28].

Appropriate antibiotic treatment is critical for better clinical outcomes. However, inappropriate antibiotic use is a common global problem that leads to antibiotic resistance, high treatment costs, and poor treatment outcomes. Hence, evaluating the appropriateness of antibiotic therapies and factors influencing these aspects is essential to designing an effective antibiotic stewardship program tailored to our setting. There is limited prior research on antibiotic appropriateness for pediatric pneumonia in northwest Ethiopia. This new study seeks to address these gaps by comprehensively assessing antibiotic appropriateness and its determinants among pediatric pneumonia patients in selected specialized hospitals in this region.

## Methods

### Study setting and period

a hospital-based prospective follow-up study was conducted among pediatric pneumonia patients attending between June 1, 2024, and September 2024, at the pediatric ward of University of Gondar Comprehensive Specialized Referral Hospital (UoGCSH), Debre Markos Comprehensive Specialized Hospital (DMCSH), and Felege Hiwot Comprehensive Specialized Hospital (FHRH).

### Study design

A multicenter institution-based prospective follow-up study was used.

### Population

patients included in the study were age ≤ 18, patients diagnosed with pneumonia by their physician (e.g., clinical symptoms, radiological findings) during their hospitalization, and patients or guardians who provided written informed consent for participation in the study. We exclude children who have no guardian and children whose pertinent information cannot be fully retrieved from either the patient’s guardian or his/her chart.

### Sample size and sampling technique

**Fig. 1:**
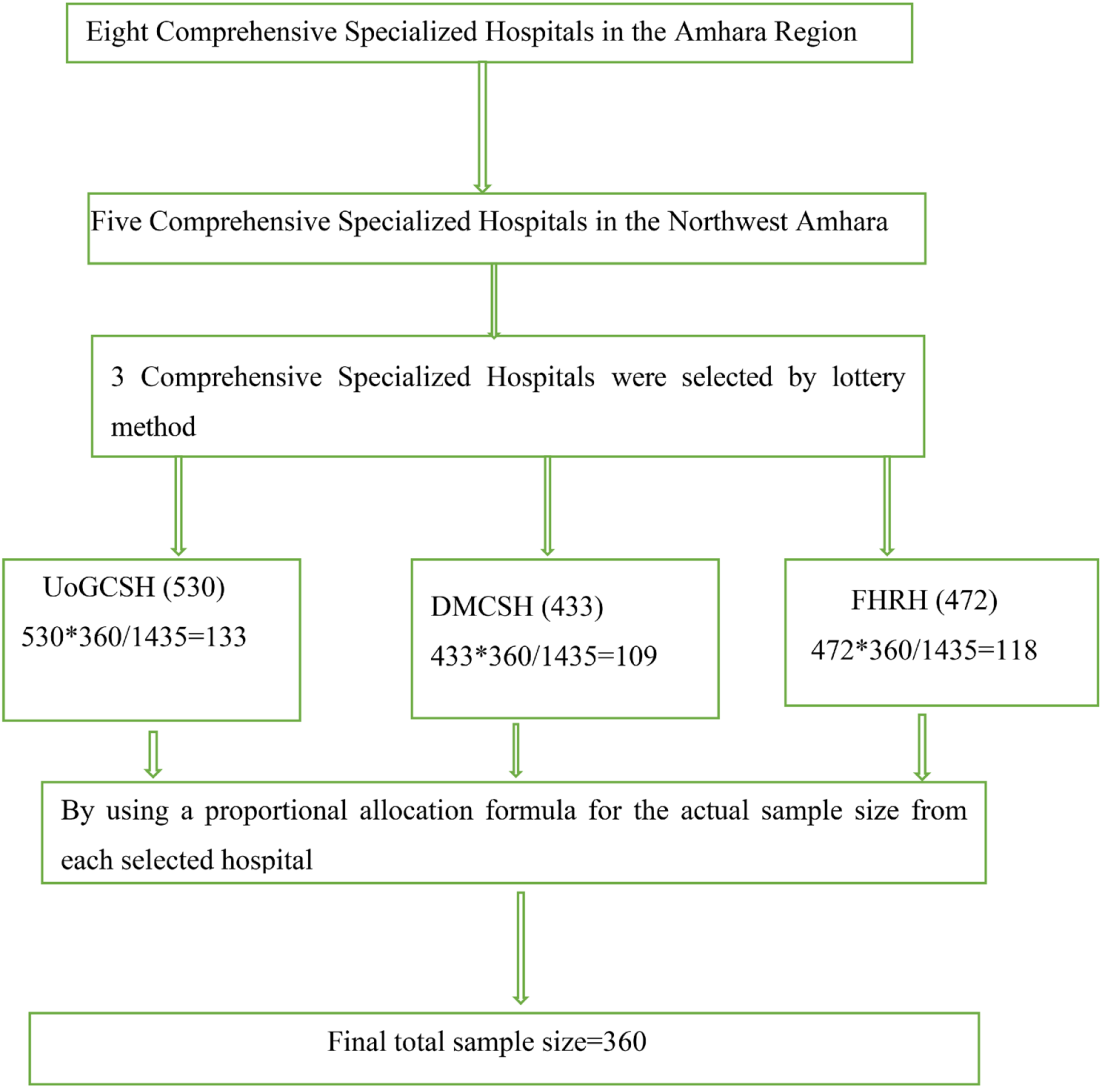
Sampling procedure flow charts on the appropriateness and its determinants among pediatric pneumonia patients in selected comprehensive specialized hospitals in Northwest Amhara, 2024

The sample size of participants for this study was calculated using the Cochran formula for categorical variables. A previous study at Tibebe Ghion Specialized Hospital, Ethiopia, reported that the inappropriate antibiotic use (the primary outcome in our study) was 30.8% [29]. The minimum sample size required for this study was 328 based on the standard normal distribution (Z = 1.96) with a confidence interval of 95% and a margin of error of 0.05. Finally, 360 patients were included after adjusting for a possible non-respondent rate of 10%. Since this research was conducted in three hospitals, the sample size was allocated proportionally to each participating hospital (Fig. 1). All patients admitted to the pediatric ward of the selected hospitals were consecutively included in the study, provided that they were available at the time of the study and fulfilled the inclusion criteria.

### Operational definition/definition of terms

#### Appropriateness of antibiotic treatments

Appropriate antibiotic use means that the patients receive the appropriate drug at the right time in adequate doses and duration to meet the individual requirements [30].

### Data collection tool and procedure

The data collection tool was adapted from available literature (supplementary file). A medical chart review was conducted to get data on diagnosis methods, types of disease, and prescribed antibiotics. The collected data were checked, assessed, and classified based on the Ethiopian national guideline 2021 and the Infectious Disease Society of America (IDSA) guidelines [6, 23, 30–34]. Appropriateness was evaluated based on four criteria: drug selection, dose, frequency, and duration, in accordance with the Ethiopian National Standard Treatment Guideline (2021) and the Infectious Diseases Society of America (IDSA) guidelines. A prescription was considered inappropriate if it failed to meet at least one of these criteria.

### Data quality control

To ensure data quality, careful preparation was undertaken before the actual data collection. Data collectors and supervisors received training on the study objectives, the content of the checklist, data collection procedures, and maintaining confidentiality and privacy. The structured checklist was adapted from previous studies and pretested on 20 patients at UGSCH before the main data collection. Based on the findings from the pretest, necessary modifications were made to improve the clarity, consistency, and overall quality of the data collection process. Three BSc nurses and three pharmacists collected the data. One supervisor was recruited for continuous supervision of the data collectors. The principal investigator closely supervised the data collection daily. At the end of each data collection day, the principal investigator checked the completeness of the filled questionnaire information to ensure its quality.

### Data entry and analysis

After coding, the data were entered into Epi-Data version 4.6, then exported, cleaned, and analyzed using SPSS version 26. Descriptive statistics were performed and presented with narration, tabulation, and graphical presentation. Normality, outliers, and multicollinearity of the variables were checked before running the bivariate and multivariate binary logistic regression analysis. Bivariate binary logistic regression analysis was conducted to identify candidate variables, and those with a p-value ≤ 0.25 were included in the multivariate logistic regression model to control for potential confounders. Model fitness was assessed using the Hosmer–Lemeshow goodness-of-fit test, and the model was considered adequate (*p* > 0.05). A multicollinearity test was performed to see the correlation between independent variables using the variance inflation factor (VIF), and no variables were observed with a VIF of > 1.5, indicating the non-existence of multicollinearity among the variables in this study. Finally, statistically significant variables were established at a p-value of 0.05 in a multivariate logistic regression model, and an adjusted odds ratio (AOR) with a 95% confidence interval was reported to measure the strength of association.

### Ethics approval and consent to participate

The proposal was submitted to the Department of Clinical Pharmacy, and ethical clearance was obtained from the University of Gondar Department of Clinical Pharmacy, Research and Ethical Review Committee, with reference number SOPS/285/2016 Ethiopian calendar. A permission letter was obtained from the department for each study setup. The nature of the study was fully explained to the study participants, and written informed consent was obtained. This study was done in compliance with the Declaration of Helsinki. Furthermore, to maintain the confidentiality of the responses, no personal identifiers were included in the questionnaires. Additionally, the study participants’ right to refuse participation was communicated to both participants and their caregivers at any time.

## Result

### Socio-demographic characteristics

A total of 360 pediatric pneumonia patients were included in the study. Of these, 212 (58.9%) were male, and most patients were in the age range of < 5 (74.7%). The median age of the study population was 1.5 years with an interquartile range of 5.25 years. Regarding vaccination status, 277 (76.9%) patients were fully vaccinated. In terms of referral sources, 227 (63.1%) patients were referred from government health facilities. Various healthcare providers wrote prescriptions; more than half of 193 (53.9%) were pediatric residents (Table 1).

**Table 1:** Socio-demographic characteristics of study participants (*N* = 360)

Variable	Category	Frequency (%)	Median age	IQR
Age (years)	< 5 5–10 10–18	269 (74.7) 56 (15.6) 35 (9.7)	1.5	5.25
Sex	Male Female	212 (58.9) 148 (41.1)		
Vaccination status	Fully Vaccinated Partially vaccinated Not vaccinated	277 (76.9) 15 (4.2) 68 (18.9)		
Referral sources	Government health center Private health center No referral	227 (63.1) 60 (16.7) 73 (20.3)		
Prescriber qualification	Pediatrician Pediatric resident General practitioner	9 (2.5) 193 (53.9) 157 (43.6)		

### Clinical characteristics and laboratory findings of participants

**Fig. 2:**
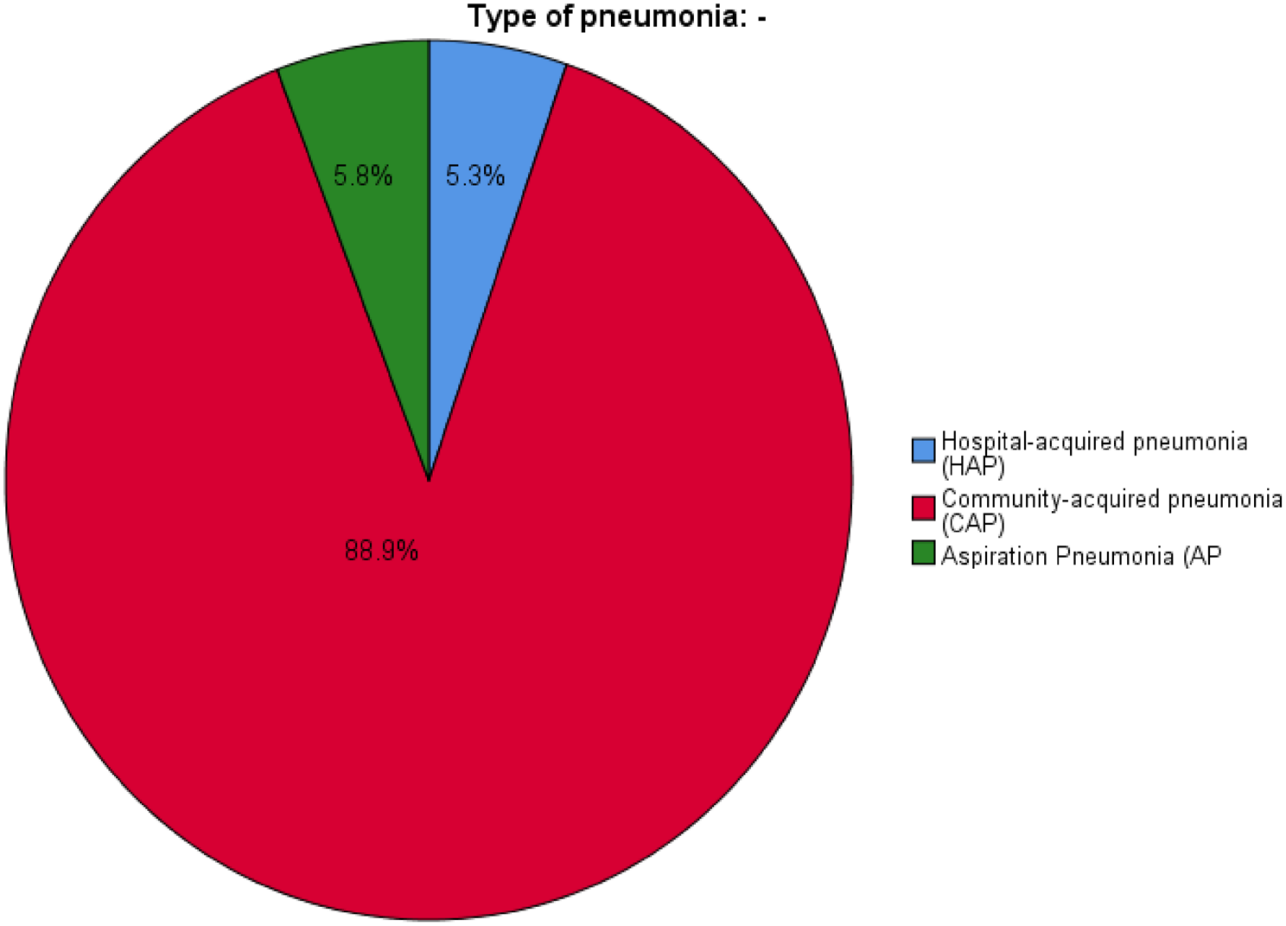
Types of pneumonia among pediatric patients admitted with pneumonia in selected comprehensive specialized hospitals in the Northwest Amhara pediatric ward

Among 360 pediatric pneumonia patients included in the present study, 320 (88.9%) had community-acquired pneumonia (Fig. 2).

Hyperactive airway disease (26.4%), severe acute malnutrition (25.1%), and congestive heart failure (18.9%) emerged as the most frequent comorbidities (Table 2).

The majority of patients, 263 (73.1%), had a hospital stay of ≤ 7 days.

Clinical findings showed vomiting (17.8%), sputum production (8.1%), loss of appetite (84.4%), and grunting (21.1%). Documented vital signs in patients included fever (88.6%), tachycardia (26.4%), decreased oxygen saturation (73.1%), and tachypnea (26.7%). Laboratory results indicated leukocytosis (60.6%), reduced hemoglobin (6.4%), and elevated neutrophil counts (81%) (Table 3).

**Table 2:** Pediatric pneumonia patients’ specific comorbidity and their frequency in selected comprehensive specialized hospitals in Northwest Ethiopia, June-September 2024 (*n* = 159)

Specific comorbidity	Frequency (%)
Hyperactive airway disease	42(26.4)
Severe acute malnutrition	40(25.1)
Congestive heart failure	30(18.9)
Acute gastroenteritis	9(5.7)
Anemia	8(5.0)
Moderate acute malnutrition	8(5.0)
Asthma	5(3.1)
Acute respiratory distress syndrome	3(1.9)
Seizure	3(1.9)
Diabetic ketoacidosis	2(1.3)
Nephrotic syndrome	2(1.3)
Pertussis	2(1.3)
Ricket	2(1.3)
Allergic dermatitis	1(0.6)
Hypothermia	1(0.6)
Hypothyroidism	1(0.6)

**Table 3:** Clinical characteristics and laboratory findings of study participants (*N* = 360)

Variable	Category	Frequency (%)
Past medical history	Yes	65 (18.1)
URTI	Yes	135 (37.5)
Vomiting	Yes	64 (17.8)
Sputum production	Yes	29 (8.1)
Fever	Yes	319 (88.6)
Loss of appetite	Yes	304 (84.4)
Grunting	Yes	76 (21.1)
Temperature	Normal Febrile Hypothermia	48 (13.3) 292 (81.1) 20 (5.6)
Heart rate	Normal Tachycardia	265 (73.6) 95 (26.4)
Oxygen saturation	Normal Decreased	97 (26.9) 263 (73.1)
Respiratory rate	Normal Tachypnea	264 (73.3) 96 (26.7)
WBC	Normal Leukocytosis	142 (39.4) 218 (60.6)
Hemoglobin	Normal Decreased	337 (93.6) 23 (6.4)
Neutrophil count (%)	Normal Increased	66 (19) 281 (81)
Length of hospital stay	≤ 7 days > 7 days	263 (73.1) 97 (26.9)

### Prescribed antibiotics, antibiotic appropriateness, and related information

All patients in the study received empirical antibiotic therapy. The most commonly prescribed antibiotic was ceftriaxone, given to 137 (38.1%) patients. Crystalline penicillin was the second most frequently used, prescribed to 120 (33.3%) patients.

A total of 138 patients (38.3%) received antibiotics considered inappropriate for their condition, with the primary reason being incorrect drug selection (42.9%) (Table 4).

**Table 4:** Antibiotic regimen pattern and appropriateness for pediatric pneumonia among participants (*N* = 360)

Name of antibiotics		Frequency (%)
Ceftriaxone		137 (38.1)
Crystalline penicillin		120 (33.3)
Ampicillin + Gentamicin		22 (6.1)
Ceftriaxone + Vancomycin		22 (6.1)
Ceftriaxone + Metronidazole		21 (5.8)
Ceftazidime + Gentamicin		10 (2.8)
Ceftriaxone + Azithromycin		7 (1.9)
Ceftriaxone + vancomycin + Metronidazole		6 (1.7)
Ceftriaxone + Ampicillin		3 (0.8)
Amoxicillin + Azithromycin		3 (0.8)
Ampicillin + Gentamicin + Azithromycin		2 (0.6)
Amoxicillin + Metronidazole		2 (0.6)
Ceftriaxone + Vancomycin + Ceftazidime		1 (0.3)
Ceftriaxone + Ampicillin + Gentamicin		1 (0.3)
Ceftriaxone + Crystalline penicillin		1 (0.3)
Ceftriaxone + Vancomycin + Gentamicin		1 (0.3)
Ampicillin + Gentamicin + Metronidazole		1 (0.3)
Total antibiotic use	Single medication	275 (76.5)
	Double medications	73 (20.3)
	Multiple medications	12 (3.3)
Inappropriate Antibiotic	No	222 (61.7)
	Yes	138 (38.3)
Cause of inappropriateness	Incorrect drug selection	67(42.9)
	Inappropriate duration	47(30.1)
	Inappropriate dose	34(21.8)
	inappropriate dosing frequency	8(5.1)

### Factors associated with inappropriate antibiotic use

In multivariate analysis, independent predictors of inappropriate antibiotic use were prescriber qualification with a pediatrician, prescriber qualification with a pediatric resident, and comorbidity. Prescriptions written by pediatricians (AOR = 5.008; 95% CI: 1.23–20.43; *p* = 0.025) and pediatric residents (AOR = 4.076; 95% CI: 2.49–6.67; *p* < 0.000) were more likely to be inappropriate compared with those from the reference group. In addition, children with comorbid conditions had a higher likelihood of receiving inappropriate antibiotic therapy (AOR = 1.717; 95% CI: 1.062–2.777; *p* = 0.027) (Table 5).

**Table 5:** Bivariate and multivariable logistic regression analysis of factors associated with inappropriate antibiotic use among pediatric pneumonia patients

Variable	Category	Inappropriate antibiotic use			COR		AOR	*p*-value
		No	Yes					
Prescriber qualification	Pediatrician	4	5		4.735 (1.204–18.826)		5.008 (1.228–20.425)	0.025
	Pediatric resident	93	100		4.073 (2.530–6.558)		4.076 (2.492–6.667)	0.000
	General practitioner	125	33		1		1	
Comorbidity	No	147	72		1		1	
	Yes	75	66		1.797 (1.163–2.775)		1.717 (1.062–2.777)	0.027
Past medical history	No	190	105		1		1	
	Yes	32	33		1.866 (1.086–3.207)		1.661 (0.918–3.008)	0.094
Previous antibiotic use within 90 days	No	181	105		1		1	
	Yes	41	33		1.387 (0.827–2.328)		1.281 (0.730–2.246)	0.388
Number of antibiotics	1	176	99		1		1	
	2	41	32		1.388 (0.822–2.343)		1.003 (0.568–1.769)	0.992
	> 2	5	7		2.489 (0.770–8.049)		1.677 (0.490–5.738)	0.410

## Discussion

This study assessed the antibiotic use appropriateness and its determinant factors among pediatric pneumonia patients in Northwest Amhara, Ethiopia. The determinant factors independently associated with inappropriate antibiotic use were prescriber qualification and comorbidity.

All patients received an empirical antibiotic therapy, which is consistent with reports from other Ethiopian settings, such as Harar town public hospital, Eastern Ethiopia [13], and Addis Ababa, TASH) [31]. This likely reflects limited access to microbiological diagnostic facilities, necessitating reliance on empirical treatment.

Ceftriaxone was the most commonly prescribed antibiotic, followed by crystalline penicillin. This finding (ceftriaxone) is higher than the 22.2% reported in Addis Ababa [35] and 22.9% in Harar [13] but lower than the 50.5% observed in studies conducted in Nekemte [36] and 45.9% in Adigrat [6]. The variation in ceftriaxone usage across these settings may reflect differences in prescribing practices, patient demographics, or the prevalence of conditions requiring ceftriaxone. Therefore, the results of this study call for the implementation of antimicrobial stewardship programs to manage the use of ceftriaxone and other antimicrobials to minimize the development of antimicrobial resistance.

The level of inappropriate antibiotic use observed in this study was higher than that reported in some settings, such as eastern Ethiopia (16.74%) [13] and Australia (24.74%) [37],. The lower inappropriate antibiotic use in Eastern Ethiopia compared to our study could be because of the complexity of diagnosing and treating pneumonia in children, which might lead to inappropriate antibiotic use, since the study in Eastern Ethiopia includes all age groups. Healthcare systems in Australia are generally well-resourced with advanced diagnostic tools, and there is also rapid access to microbiological testing, along with strong antimicrobial stewardship programs. These factors would probably contribute to a high level of adherence to antibiotic guidelines.

However, the prevalence observed in this study is lower than findings from Uganda (75.1%) [12] and Pakistan (90.2%) [10]. The Ugandan study focused specifically on children under five with severe pneumonia, which might lead to more empirical antibiotic use because of diagnostic challenges or urgency in treating critically ill children, while our study focused on pediatric pneumonia (up to 18 years). Differences in healthcare settings and treatment practices may also explain the observed variation.

The large discrepancy between the inappropriateness of antibiotic use in our study and that of Pakistan can be attributed to factors such as differences in study design, population, and the guidelines used in assessing appropriateness. Our study’s prospective design allowed for real-time data collection. Our study focused on pediatric patients up to 18 years of age, whereas in Pakistan, it focused on children under 5 years.

Inappropriate drug selection emerged as the leading cause of inappropriate antibiotic use, followed by inappropriate duration, inappropriate dose, and inappropriate frequency. This was in line with studies done in Jimma [38] and Adigrat [6], which showed inappropriate drug selection, dose, frequency, and duration, were the commonly observed inappropriate antibiotic uses. In Nekemte [39] inappropriate dose, duration, and frequency were the causes of inappropriateness in antibiotic use. Another study in Malaysia [40] showed that inappropriate drug selection, dose, duration, and route were causes of inappropriate antibiotic use.

In comparison, studies in other settings have reported varying patterns of causes. For instance, a study conducted in Harar [13] identified only incorrect drug selection and prolonged duration as major contributors to antibiotic inappropriateness, with no significant problem reported regarding dose or frequency. This could reflect better adherence to guideline-recommended dose or dosing intervals in those settings.

These findings demonstrate the variability in inappropriate antibiotic use across different settings, emphasizing the influence of healthcare infrastructure, guideline adherence, and resource availability. Understanding these contextual factors is crucial for addressing the underlying causes of inappropriate antibiotic use, particularly in settings with alarmingly high rates.

Our study identified the independent predictors of inappropriate antibiotic use as prescriber qualification and comorbidity. There are plausible explanations for the influence of the prescriber qualification on inappropriate antibiotic use. These include clinical experience, continuing medical education, and attitude towards and/or knowledge of antibiotic resistance [41, 42]. The lack of institutional guidelines and antimicrobial stewardship programs may be another potential reason for inappropriate antibiotic prescribing [43]. In addition, marketing and promotional activities by pharmaceutical companies may influence physicians’ prescribing patterns [44]. Prescribers themselves are also subject to several pressures, including the lack of standardized national guidelines, the unavailability of key antibiotics in the hospital pharmacies, poor financial resources of the families, and pressure from caregivers to prescribe inappropriate antibiotics [45]. Overreliance on clinical judgment rather than guidelines may also contribute to inappropriate prescribing.

The presence of comorbidity was another predictive factor for inappropriate antibiotic use in children. This is supported by the studies conducted in Ethiopia (Harar) [13], the Netherlands [46], and the United States, which show that comorbidity doubles a child’s risk of being among the top 20% of antibiotic recipients, and this could lead to inappropriate antibiotic use [47]. Comorbid conditions often may complicate the clinical presentation, leading to diagnostic uncertainty and potential overuse or misuse of antibiotics [48]. One possible explanation is that patients with comorbidities are more likely to present with atypical or severe symptoms [48], which may prompt clinicians to prescribe broad-spectrum antibiotics empirically, even when not indicated. Additionally, managing comorbid conditions often involves multiple healthcare providers, increasing the risk of fragmented care and inconsistent adherence to antibiotic guidelines [49].

These findings underscore the importance of developing targeted interventions, such as enhanced diagnostic tools and clinician training, to improve antibiotic prescribing practices for patients with comorbidities.

## Limitations of the study

Although the prospective design of this study provided for a focused and consistent approach to data collection, it is essential to acknowledge the real-world constraints that shaped our findings. First, this research was conducted against the ongoing conflict in the Amhara region. We recognize that such instability does more than disrupt services; it creates significant barriers for families trying to reach the hospital. Consequently, our results represent a specific group of patients who were able to access care despite these hardships, which may limit the representativeness of our findings to the broader population.

Second, our assessment of “appropriateness” was affected by the clinical realities of a resource-limited setting. In the absence of routine microbiological testing and cultures, clinicians must trust their best judgment through empirical guidelines. While we measured adherence to these protocols, we could not account for the specific pathogens or resistance patterns that might have justified a deviation from standard care. Finally, the study did not include healthcare providers’ perspectives on barriers to guideline adherence, which could have provided valuable context for understanding the challenges in clinical practice.

## Conclusion

Our findings indicate that over one-third of children hospitalized with pneumonia received inappropriate antibiotic therapy, primarily due to incorrect drug selection. The fact that specialized prescribers and the presence of comorbidities were linked to inappropriate antibiotic use suggests that even experienced clinicians face challenges in aligning complex cases with standard protocols. These results highlight a clear, human need for improved adherence to national treatment guidelines. Rather than broad systemic changes, we recommend focused support and practical training for clinical staff to ensure that every child receives the most effective, guideline-based care, even in resource-limited settings.

## Electronic Supplementary Material

Below is the link to the electronic supplementary material.

Supplementary Material 1

## Data Availability

The data sets used and/or analyzed during the current study are available from the corresponding author on reasonable request.

## Abbreviations

OR: Adjusted odds ratio
COR: Crude odds ratio
CI: Confidence interval
CAP: Community-Acquired Pneumonia
UoGCSH: University of Gondar Comprehensive Specialized Referral Hospital
DMCSH: Debre Markos Comprehensive Specialized Hospital
FHRH: Felege Hiwot Comprehensive Specialized Hospital (FHRH)
IDSA: Infectious Disease Society of America
SPO_2_: Peripheral Capillary Oxygen Saturation
URTI: Upper respiratory tract infection
