# Supplementary material for: Antibiotic use appropriateness and its determinant factors among pediatric pneumonia patients in selected comprehensive specialized hospitals in Northwest Amhara, 2024: a prospective follow-up study
Source: BMC Infect Dis. 2026 Apr 28;26:1145. doi: 10.1186/s12879-026-13447-8 (PMC13267253; doi:10.1186/s12879-026-13447-8)
Supplement: Supplementary file 1 — Supplementary Material 1 [file 12879_2026_13447_MOESM1_ESM.docx]

Antibiotic Use Appropriateness and Its Determinant Factors among Pediatric Pneumonia Patients in Selected Comprehensive Specialized Hospitals in Northwest Amhara, 2024: A Prospective Follow-up Study

**Data collection format**

**Code __________**

1. Demographic characteristic
   1. Age: ____________
   2. Sex: ____________
   3. weight: _________
   4. Card No: ____________
   5. Referred from: ________
   6. Date of admission: ____
2. Past medical history: ___________________________
3. Vaccination status______________________________
4. Does the patient have a recent antibiotic medication use history within 90 days? Yes No
5. If yes for question no 4 specify which antibiotic_______________________________
6. Date of pneumonia diagnosis: _____________________________________________
7. Admission diagnosis: ____________________________________________________
8. Type of pneumonia: -
9. Hospital-acquired pneumonia (HAP)
10. Community-acquired pneumonia (CAP)
11. Aspiration Pneumonia (AP)

Other specify: ______________________

1. Factors that predispose pneumonia (You can mark “√” one or more risk factors)
   1. Upper respiratory tract infections
   2. Corticosteroid therapy
   3. Recent influenza infection
   4. Pre-existing lung disease
   5. unimmunized
   6. Others, Specify________________
2. Change in diagnosis (if any): ___________________
   1. Date of change: ______________
   2. Reason for change (Ask the physician in charge):
      1. Additional Investigation data obtained

- - 1. New clinical signs and symptoms observed
    2. Senior consultation (specify) ______________
    3. Other (specify)_________________________

1. Is there a microbiologic test (gram stain and/or culture and sensitivity)?
   1. Yes

- 1. No

1. Date of sampling for the microbiologic test_________________
2. If yes to question 11, microbiologic study results:
   1. Source: ____________gram stain: ___
   2. Causative pathogen (s): __________
   3. Susceptibility data: _____________
   4. Time of culture collection: _______
3. If no to question number 11, what was the reason (Ask the physician in charge)?
   1. There is no institutional guidance that recommends testing
   2. No well-equipped microbiology lab
   3. This is the usual practice
   4. Other reasons, specify-----
4. Initial antibiotic therapy? Empiric definitive
5. Initial Empiric/definitive Antibiotic therapy:

| Drug name | Dose and frequency | Route | Duration | Indication |
| --- | --- | --- | --- | --- |
|  |  |  |  |  |
|  |  |  |  |  |
|  |  |  |  |  |
|  |  |  |  |  |

Any missed dose and reason________________________________________________

1. Prescribing physician (empiric or definitive):
   1. pediatrician
   2. pediatric resident
   3. General practitioner
   4. other specify _________________
2. Which guideline is used to prescribe the above (q. 16) medication (s) to the patient?
   1. FMHACA ‘s STG
   2. Institutional guideline/protocol
   3. Other international guideline (s) (specify): _____________
   4. Reference Book (s) (specify)________________________
   5. Other (specify)___________________________________
3. Is there a change in antibiotic agent (s) or regimen?
   1. Yes
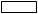

   2. No
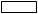

4. If yes to the above question, mention the following information:

First-time change

| Drug name | Dose and frequency | Route | Duration | Indication |
| --- | --- | --- | --- | --- |
|  |  |  |  |  |
|  |  |  |  |  |
|  |  |  |  |  |
|  |  |  |  |  |

Any missed dose and reason________________________________________________

Second-time change

| Drug name | Dose and frequency | Route | Duration | Indication |
| --- | --- | --- | --- | --- |
|  |  |  |  |  |
|  |  |  |  |  |
|  |  |  |  |  |
|  |  |  |  |  |

Any missed dose and reason________________________________________________

Third time change

| Drug name | Dose and frequency | Route | Duration | Indication |
| --- | --- | --- | --- | --- |
|  |  |  |  |  |
|  |  |  |  |  |
|  |  |  |  |  |
|  |  |  |  |  |

Any missed dose and reason________________________________________________

1. Reason for change:
   1. Poor response
   2. Inadequate selection
   3. Side effects of antimicrobials
   4. Drug shortage
   5. Not Defined
   6. Change in diagnosis
   7. Other specify: _____________________
2. Prescribing physician (the physician that changes the regimen):
   1. Pediatrician
   2. Pediatric resident
   3. General practicioner

other (specify)_______

Other (specify)___________________

1. Length of hospital stay_______
2. Clinical signs and symptoms: Cough Sputum production Fever Other(specify)___

1. Vital signs:

| V/s start from pneumonia dx | **Date/Time** | | | | | | | | | | | | |
| --- | --- | --- | --- | --- | --- | --- | --- | --- | --- | --- | --- | --- | --- |
|  | Normal range |  |  |  |  |  |  |  |  |  |  |  |  |
| T(C)ͦ | 36.5-37.2 |  |  |  |  |  |  |  |  |  |  |  |  |
| BP(mmHg) |  |  |  |  |  |  |  |  |  |  |  |  |  |
| PR (per min) |  |  |  |  |  |  |  |  |  |  |  |  |  |
| RR (per min) |  |  |  |  |  |  |  |  |  |  |  |  |  |
| SaO_2_ on atm. | >92% |  |  |  |  |  |  |  |  |  |  |  |  |
| SaO_2_ on oxy. | >95% |  |  |  |  |  |  |  |  |  |  |  |  |

- 1. Lab findings: start from pneumonia dx and the last result when medication is stopped

| **Tests** | |  | **Results** | | | |
| --- | --- | --- | --- | --- | --- | --- |
| **Parameters** | | **Ranges** | **Initial value** | **End of Rx** | **Remark** | |
|  | |  |  |  |  |  |
| **CBC/FBC** | WBC |  |  |  |  |  |
|  | Neutrophils% |  |  |  |  |  |
|  | Hb test |  |  |  |  |  |
